# Cleavage of E-Cadherin by Matrix Metalloproteinase-7 Promotes Cellular Proliferation in Nontransformed Cell Lines via Activation of RhoA

**DOI:** 10.1155/2010/530745

**Published:** 2010-06-10

**Authors:** Conor C. Lynch, Tracy Vargo-Gogola, Lynn M. Matrisian, Barbara Fingleton

**Affiliations:** ^1^Department of Cancer Biology, Vanderbilt University School of Medicine, 734 PRB 2220 Pierce Ave, Nashville, TN 37232, USA; ^2^Department of Orthopaedics and Rehabilitation, Vanderbilt University School of Medicine, Nashville, TN 37232, USA; ^3^Department of Biochemistry and Molecular Biology, Indiana University School of Medicine, South Bend, IN 46617, USA

## Abstract

Perturbations in cell-cell contact machinery occur frequently in epithelial cancers and result in increased cancer cell migration and invasion. Previously, we demonstrated that MMP-7, a protease implicated in mammary and intestinal tumor growth, can process the adherens junction component E-cadherin. This observation leads us to test whether MMP-7 processing of E-cadherin could directly impact cell proliferation in nontransformed epithelial cell lines (MDCK and C57MG). Our goal was to investigate the possibility that MMP-7 produced by cancer cells may have effects on adjacent normal epithelium. Here, we show that MMP-7 processing of E-cadherin mediates, (1) loss of cell-cell contact, (2) increased cell migration, (3) a loss of epithelial cell polarization and (4) increased cell proliferation via RhoA activation. These data demonstrate that MMP-7 promotes epithelial cell proliferation via the processing of E-cadherin and provide insights into the molecular mechanisms that govern epithelial cell growth.

## 1. Introduction

The matrix metalloproteinase (MMP) family of zinc-dependent enzymes comprises 23 members in man [1]. Although MMPs are typically thought of as degradative enzymes with the ability to cleave extracellular matrix proteins such as collagen and fibronectin, recent literature suggests that their dominant role is actually as signal-altering molecules [2]. MMP-7, in particular, has several in vivo-verified substrates, none of which are typical extracellular matrix proteins [3]. These nonmatrix-degrading functions of MMPs vastly expand the ways in which they can contribute to various pathologies, including tumor progression. In previous studies we have identified several growth and death factors as substrates for MMP-7 that affect tumor behavior [4–7]. In addition, we identified the adhesion molecule E-cadherin as an MMP-7 substrate and showed that one of the products of E-cadherin cleavage, the 80 kDa ectodomain, could promote invasive activity in a paracrine fashion [8]. These previous studies were all performed in transformed cells. This led us to question whether the functions of MMP-7 are similar in nontransformed cells. This is an important question since MMP-7 is a secreted proteinase, and it is likely that its release from cancer cells could affect adjacent normal tissue.

E-cadherin is the prototypical epithelial adhesion molecule [9, 10]. One of its functions is to sense cell:cell contact and thus limit density. E-cadherin can be modulated in many ways, including methylation of the promoter resulting in downregulation [11], gene mutation as seen in familial gastric carcinoma [12], downregulation of expression by binding of transcription factors such as Snail and Slug to the promoter [13], and extracellular proteolysis [14]. This last process results in the production of the afore-mentioned 80 kDa ectodomain fragment that is detectable in the serum and urine of cancer patients and has been proposed as a biomarker [15]. Several proteinases are known to cleave E-cadherin including MMPs-3 and -7 [8, 16], ADAMs-10 [17] and -15 [18], cathepsins B, L, and S [19], and the serine proteinases plasmin [20] and kallikreins-6 [21] and -7 [22]. It is typically thought that such E-cadherin processing results in cells with enhanced migratory potential; however functions of E-cadherin other than adhesion are also affected by proteolytic events. Previous studies have suggested that downregulation of E-cadherin in tumor cells leads to a reduction in levels of the cyclin-dependent kinase inhibitor p27^kip1^ and ultimately enhanced proliferation [23]. Here we use nontransformed epithelial cell lines to test whether proliferation is altered when E-cadherin is processed by MMP-7. We demonstrate that E-cadherin cleavage by MMP-7 initiates an alternative proliferative pathway that bypasses p27 but results in enhanced RhoA activity and increased cyclin D1 levels. Thus, in addition to enhanced migratory ability, a predominant result of MMP-7-mediated E-cadherin cleavage is loss of contact inhibition and an increase in proliferation indicating that MMP-7 cleavage of E-cadherin can regulate growth potential of nontransformed cells.

## 2. Materials and Methods

### 2.1. Cell Lines and Culture Conditions

Madin Darby Canine Kidney (MDCK) and the mammary gland cell line derived from a C57BL/6 mouse (C57MG) cells have been described [24, 25]. Retroviral transduction was used to generate clones of C57MG cells stably expressing MMP-7 as previously described [4]. All cells were maintained in DMEM containing 10% fetal calf serum at 37°C, with a 5% CO_2_-humidified atmosphere. For experiments where serum-free conditions were required, the growth medium was removed, and cells were rinsed with PBS and then refed with OPTI-MEM medium (Invitrogen, Carlsbad, CA) containing no serum. To generate polarized monolayers of MDCK and C57MG cells, 2 × 10^6^ cells were plated on the upper surface of 0.4 *μ*m Transwell filters (Corning, Lowell, MA) in 6-well plates. The resistance of the cultures was measured daily using an epithelial volt ohmmeter (World Precision Instruments, Sarasota, FL). The MDCK cell layer was considered polarized when the trans-epithelial resistance was greater than 200 *Ω* · cm^2^. For all experiments where recombinant MMP-7 (Calbiochem/EMD Chemicals, Gibbstown, NJ) was added to cultures, a concentration of 100 ng/mL was used.

### 2.2. Cell Growth and Proliferation Assays

Growth rates of the C57MG clones were determined by plating 10^4^ cells in 6-well plates (for low density) or 5 × 10^4^ cells in 12-well plates (for high density), and viable cells were counted using a hemocytometer every two days for 10 days. Further analysis of the C57MG clones was performed using the Cell Titer 96 Aqueous Nonradioactive cell proliferation assay (Promega, Madison, WI), according to the instructions provided by the manufacturer. For these assays, 5 × 10^3^ cells were seeded per well in 96-well plates. The matrix metalloproteinase inhibitor BB-94 (a gift from Dr Peter Brown, British Biotech plc, Oxford, UK) was added at time of seeding to replicate wells at either 2.5 or 5 *μ*M to determine the contribution of matrix metalloproteinase activity to proliferation. Control wells received equivalent volumes of the vehicle, dimethylsulfoxide. To test the contribution of E-cadherin, an E-cadherin blocking antibody (Decma-1, Sigma, St Louis, MO) or control rat IgG (Sigma) was added to replicate wells at a concentration of 40 mg/mL. Prior to adding the antibodies, cells had been serum-starved for 24 hours, then incubated with 5 mM EGTA (Sigma) for 30 mins to disrupt adherens junctions. Thymidine incorporation assays were used as an indicator of proliferation rate of polarized MDCK cells. Once polarization had been confirmed, MDCK cells were serum-starved for 24 hrs, then treated with 100 ng/mL recombinant active human MMP-7 (EMD Chemicals, Gibbstown, NJ) added to either the apical or basal compartment. A second addition of MMP-7 occurred after 18 hrs. Four hours later, the transepithelial resistances were measured, and 1.0 mCi of methyl-^3^H-thymidine (Perkin-Elmer/NEN, Waltham, MA) was then added to the basal surface of all cells. After 3 hours, the filters were washed in ice-cold 10% trichloroacetic acid (Sigma), dried and then incubated overnight in 0.2 N sodium hydroxide. Aliquots of the supernatant were then removed for scintillation counting.

### 2.3. Migration Assay

C57MG cells were removed from tissue culture flasks with 5mM EDTA. After washing with PBS, the cells were resuspended in serum-free medium and 1.5 × 10^4^ cells were placed on the upper surface of 8.0 *μ*m Transwell filters in 24-well plates (Corning). In the lower chamber, 1 mL of DMEM containing 10% FBS was used as a chemoattractant. The cells were then incubated for 6 hours at 37°C in a 5% CO_2_ humidified incubator. After incubation, the cells on the upper surface of the membrane were removed with a cotton-tipped swab. The membranes were cut out of the chamber, and the cells on the lower surface were fixed in 100% methanol at −20°C for 7 minutes and stained with hematoxylin and eosin. The membranes were then dehydrated through a series of ethanol solutions and mounted on slides using an aqueous mounting medium. Migrating cells were then viewed under the microscope at 10x magnification, and the number of migrating cells per filter was determined.

### 2.4. Western Blotting and Immunoprecipitation

For analysis of E-cadherin, cyclinD1, and p27^kip1^ expression, confluent cultures of MDCK cells treated with or without recombinant human MMP-7 were lysed in lysis buffer (0.5% NP-40, 100 mM NaCl, 50 mM Tris-HCL, pH 7.5, containing a protease inhibitor cocktail (Roche, Indianapolis, IN)), and 30 *μ*g of total protein was electrophoresed through SDS-polyacrylamide gels. For the C57MG clones, 10^4^ cells were inoculated into 6-well plates and after 8 days (the time point identified at which the clones were confluent (Figure 3(c)) cell lysates were harvested in a similar manner to those described for MDCK. Proteins were transferred to PVDF membranes for detection. Anti-p27^kip^ was used at a 1 : 2,500 dilution (clone 57, BD-Transduction, San Diego, CA), anticyclin D1 (Santa Cruz Biotechnology, Santa Cruz, CA) was used at 1 : 1000, while anti-E-cadherin was used at 1 : 500 (Decma-1, Sigma). 

For detection of soluble E-cadherin in conditioned medium from C57MG clones, E-cadherin was immunoprecipitated from 3 mls of medium that had been conditioned for 48 hours. To reduce background, samples were incubated with 100 *μ*L of protein A-sepharose (25 mg/mL stock, Amersham/GE Healthcare, Piscataway, NJ) in immunoprecipitation (IP) buffer (50 mM Tris pH 7.5, 100 mM NaCl, 0.5% NP-40) at 4°C for 1 hour. Precleared lysates were then incubated with 1 *μ*g of anti-E-cadherin (Decma-1) for 1 hour. After this step, 200 *μ*L of protein A-sepharose beads was added, and the samples were incubated overnight at 4°C. The immunoprecipitates were analyzed by Western blotting using the anti-E-cadherin antibody (Decma-1) as described above.

### 2.5. Immunofluorescence

All cell lines were plated at confluent densities (5 × 10^3^ cells/well) in an 8-well plastic chamber slide (LabTek, Thermo Fisher Scientific, Pittsburgh, PA). After 24 hours, the cells were examined for E-cadherin or *β*-catenin localization using immunofluorescence. The cells were rinsed once in PBS and were fixed for 7 minutes at −20°C in 100% methanol. The methanol was removed and the cells were washed twice in PBS for 5 minutes with shaking at room temperature. All subsequent washes were performed with shaking. The cells were then incubated for 30 minutes at room temperature using a 3% blocking solution (3% milk in PBS). The cells were then washed four times for 5 minutes with PBS. After washing, the cells were incubated with the primary antibodies (*β*-catenin: 1 : 250 dilution, BD-Transduction; E-cadherin 1 : 100, Sigma) for 1 hour at room temperature. The cells were then washed four times for 5 minutes in PBS. A Cy3-labeled antimouse secondary antibody (Invitrogen) was added at a dilution of 1 : 1000 for 30 minutes at room temperature. The slides were then washed four times for 10 minutes in PBS with shaking. After the final wash, the slides were covered with an aqueous mountant (Gel/Mount, Biomeda, Burlingame, CA), coverslipped and viewed with a fluorescent microscope.

### 2.6. RhoA Activity Assays

To assess changes in the activation status of RhoA, rhotekin pull-down assays were used as described [26]. Briefly, 10 cm plates of C57MG clones under preconfluent or confluent conditions, or MDCK cells exposed to 100 ng/mL recombinant active MMP-7 for different periods of time, were lysed in 1% NP-40, 50 mM Tris, pH 7.4, 10% glycerol, 100 mM NaCl, and 10 mM MgCl2. A rhotekin binding domain-glutathione S transferase (RBD-GST) fusion protein (vector kindly supplied by Dr Neil Bhowmick, Vanderbilt University) was precoupled to agarose-glutathione beads (Sigma) and used to adsorb the GTP-bound (active) form of RhoA from the lysates. Total RhoA levels from nonadsorbed lysates as well as the bead-adsorbed active RhoA was detected by immunoblotting using a RhoA antibody from Santa Cruz Biotechnology. As a positive control for these assays, cells were treated with lysophosphatidic acid (LPA), known to simulate Rho A activation [26], for 5 mins.

### 2.7. Statistical Analysis

All analysis was performed, and graphs generated using Prism 5 software (Graphpad software, San Diego, CA). Significance was defined at the 95% level of confidence. For all experiments, a minimum of 3 replicates per condition was used. Each experiment was repeated at least twice. For comparisons between 2 groups, Student's *t*-test was used. Nonparametric data were compared using the Mann-Whitney test. One-way ANOVA with Bonferroni or Tukey post hoc test (indicated in appropriate Figure Legends) was used for analyzing data from multiple groups. Data are shown as mean  ±  standard deviation. 

## 3. Results

### 3.1. MMP-7 Disrupts Epithelial Adherens Junctions and Promotes a Migratory Phenotype

**Figure 1:**
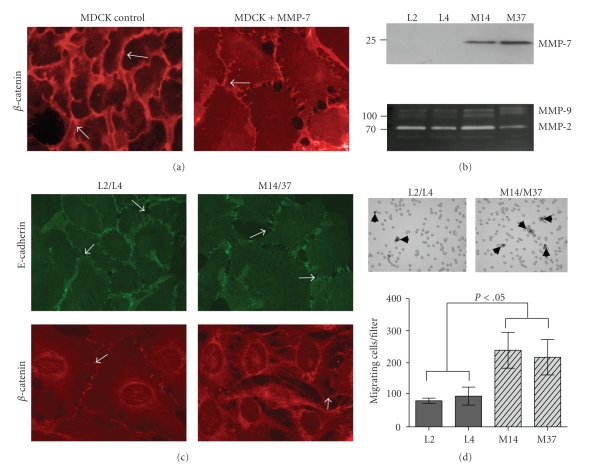
MMP-7 expression disrupts adherens junctions. (a) Representative photomicrograph (400x) of *β*-catenin immunofluorescent staining in MDCK cells in the absence or presence of exogenous MMP-7 (100 ng/mL). Arrows indicate junctional *β*-catenin. (b) Immunoblot analysis of MMP-7 (27 kDa) expression (upper panel) in empty vector control (L2 and L4) and MMP-7 (M14 and M37) transduced C57MG cell lines. Zymography (lower panel) was used to examine the expression of MMP-2 (72 kDa) and MMP-9 (92 kDa). (c) Representative photomicrographs (400x) of E-cadherin (green) and *β*-catenin (red) immunofluorescent staining (arrows) in the vector control (L2/L4) and MMP-7 (M14/37) expressing cells. Arrows indicate junctional E-cadherin and *β*-catenin. (d) The impact of MMP-7 expression on cell migration through 8 *μ*m pore membranes (arrow heads) towards 10% serum-containing media was determined. *P < .05, as determined by ANOVA followed by Tukey's post hoc test.

The MDCK cell line is a nontransformed, nontumorigenic canine kidney cell line that does not express MMP-7 endogenously and has been extensively utilized as an in vitro model to study adherens junction biology [7, 24]. Using confluent MDCK cells, we observed by immunofluorescence that the addition of exogenous MMP-7 resulted in a loss of cell-cell contact as assessed by immunofluorescent localization of *β*-catenin (Figure 1(a)). To ensure that this effect was not specific to the MDCK cell line, we employed another nontransformed, nontumorigenic mammary gland epithelial cell line, C57MG, that does not endogenously express MMP-7 [4]. To more accurately reflect the in vivo scenario of endogenous MMP-7 expression, we generated C57MG clonal cell lines that were either negative (L2, L4) or positive (M14, M37) for MMP-7 expression (Figure 1(b)). Importantly, we observed that the endogenous expression of MMP-7 in the C57MG cell line did not impact the expression of other MMPs such as MMP-2 and MMP-9. Concomitant with our observations in the MDCK cell line, the endogenous expression of MMP-7 resulted in a loss of adherens junction formation as assessed by E-cadherin and *β*-catenin immunofluorescent localization in the MMP-7 expressing clones (Figure 1(c)). Analysis of the migratory phenotype of the C57MG cell lines also demonstrated that MMP-7 expression significantly promoted migration toward serum containing media (L2; 81.3 ± 8.50, L4; 96 ± 28.0 versus M14; 239 ± 56.0, M37; 217.3 ± 55 migrating cells per filter, Figure 1(d)). These data identify that the addition of exogenous recombinant active MMP-7, or the forced expression of MMP-7, results in a disruption of the adherens junction and an increase in cellular migration in nontransformed epithelial cell lines.

### 3.2. Epithelial Cell Polarization Is Compromised in the Presence of MMP-7

**Figure 2:**
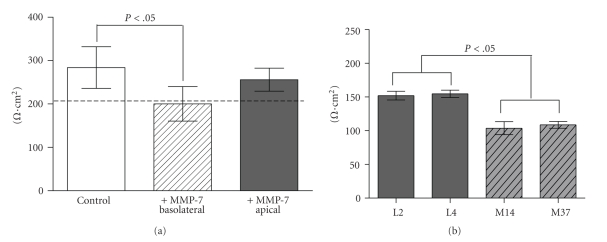
Epithelial polarization is negatively impacted by MMP-7. (a) Analysis of MDCK polarization in response to the addition of basolateral or apical exogenous MMP-7 (100 ng/mL) to the lower or upper compartment of the transwell chamber, *P < .05 as determined by ANOVA followed by Bonferroni's post hoc test. Dashed line represents the resistance level at which MDCKs are polarized [7]. (b) Analysis of C57MG cell line transepithelial resistance in response to the expression of MMP-7. Confluent (day 8) MMP-7 expressing clones (M14 and M37) growing on a 0.4 *μ*m transwell membrane never reached a resistance that was comparable to that of the empty vector control C57MG clones.

Epithelial polarization is critical for the proper cell function and the formation of tight adherens junctions are key for this process. Therefore, given that MMP-7 appeared to be mediating the disruption of the adherens junction, we examined whether addition or expression of MMP-7 impacted the ability of nontransformed epithelial cell lines to polarize. Our results show that the addition of MMP-7 to the basolateral compartment (lower chamber) of confluent MDCK cells grown on a 0.4 *μ*m membrane resulted in a loss of polarization compared to the control cells or to the addition of MMP-7 to the apical surface (Control; 284 ± 48.1 versus MMP-7-Basolateral; 200 ± 39.9 versus MMP-7-Apical; 256.0 ± 26.5 *Ω* · cm^2^, Figure 2(a)). In addition, we observed that MMP-7 expression by the C57MG clones, M14, and M37 also impacted the tightness of the monolayer formed by confluent C57MG cell lines as determined by epithelial resistance measurements (L2; 152 ± 6.48, L4; 155 ± 5.430 versus M14; 104 ± 9.67, M37; 109 ± 4.99 *Ω* · cm^2^, *P* < .05, Figure 2(b)). These data are in agreement with our observations examining the disruption of the adherens junctions by the addition or expression of MMP-7 and further perturbation of the adherens junctions negatively impacts the ability of nontransformed epithelial cells to form tight cell: cell junctions necessary for polarization.

### 3.3. MMP-7 Promotes Epithelial Proliferation In Vitro

**Figure 3:**
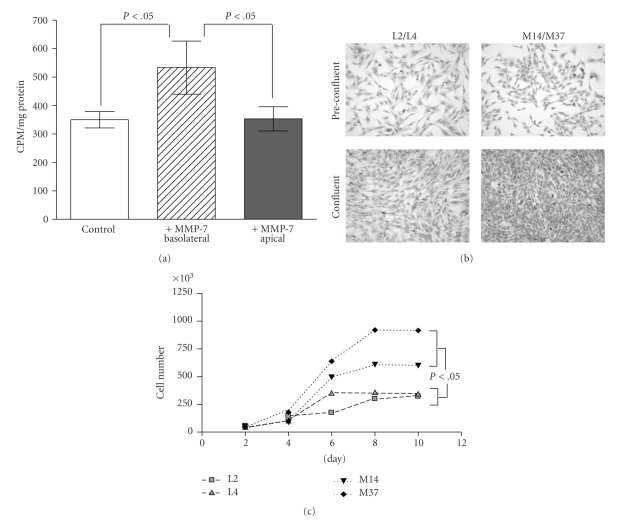
MMP-7 enhances the proliferation of nontransformed epithelial cells. (a) Analysis of proliferation using [3H]-thymidine of confluent MDCK (grown on 0.4 *μ*m transwell membrane) upon addition of exogenous MMP-7 (100 ng/mL) to the basolateral (lower chamber) or apical surface (upper chamber). *P < .05 as determined by one-way ANOVA with Bonferroni post hoc test. The values represent scintillation counts corrected for amount of protein present. (b) Representative photomicrographs of vector control (L2/L4) and MMP-7 expressing (M14/37) C57MG cells under subconfluent and confluent conditions (200x). (c) The average cell counts of vector control (L2 and L4) and MMP-7 (M14 and M37) expressing C57MG cell lines over time. ANOVA analysis followed by Bonferroni's post hoc analysis of the numbers of cells in each group at the day 10 time point revealed that the M14 and M37 cell lines reached significantly higher numbers of cells (*P* < .05).

Cell-cell contact is a negative regulator of proliferation [27]. Since MMP-7 perturbed the ability of epithelial cells to form stable adherens junctions and polarize, we tested whether the exogenous addition or expression of MMP-7 enhanced the proliferation of MDCK and C57MG cells. Using a transwell system, MDCK cells were grown to confluence, that is, contact inhibition, and we observed that the addition of MMP-7 to the basolateral surface promoted the proliferation of the MDCK cells compared to the addition of MMP-7 to the apical surface or control conditions as assessed by [3H]-thymidine corporation (Control; 350 ± 28.0 versus MMP-7-Basolateral; 533 ± 93.0 versus MMP-7-Apical; 353.3 ± 42.7 counts per minute/mg total protein as measured in scintillation counter, Figure 3(a)). These data suggest that disruption of cell:cell adhesion is sufficient to facilitate the proliferation of contact-inhibited MDCK cells. 

The effect of MMP-7 expression on the proliferation of the C57MG cell line was also determined. Interestingly, preconfluent analysis showed no morphological differences between the empty vector control and MMP-7 expressing C57MG clones, but it appeared that MMP-7 expression conferred the C57MG cells with an ability to grow to a much higher cell saturation density (Figure 3(b)). Analysis of the logarithmic growth of the cells over time using trypan blue exclusion assays revealed that the average doubling time for each of the cell lines was L2; 34.8 ± 1.00, L4; 28.1 ± 1.50 versus M14; 25.6 ± 0.50, M37; 24.1 ± 1.00 hours. Statistical analysis revealed that the MMP-7 expressing cell lines had a significantly faster doubling time (*P* = .002) and grew to significantly higher cell densities compared to the empty vector control cell lines (Figure 3(c)). These data demonstrate that the MMP-7 can promote the proliferation of nontransformed epithelial cells.

### 3.4. E-Cadherin Is Processed by MMP-7 in Nontransformed Epithelial Cell Lines

**Figure 4:**
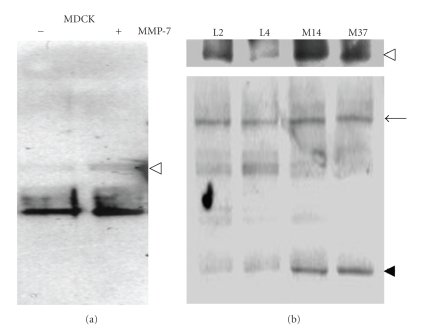
MMP-7 promotes E-cadherin processing. (a) Detection of the ectodomain of E-cadherin (80 kDa, open arrow head) in the conditioned media of MDCK cells in the presence (+) or absence (−) of exogenous MMP-7 (100 ng/mL). (b) Analysis of E-cadherin processing in the empty vector control (L2 and L4) and MMP-7 (M14 and M37) expressing cell lines. Open arrow head indicates the ectodomain of E-cadherin (80 kDa) in conditioned media, while arrow and arrow head indicate full length E-cadherin (120 kDa) and intracellular fragment of E-cadherin (40 kDa), respectively, in C57MG cell line lysates.

The adherens junction in epithelial cells contains numerous complexes that allow the formation of tight cell-cell contacts. Previously, we identified that MMP-7 was capable of processing E-cadherin in transformed cells, at the juxtamembrane region, resulting in the generation of 80 kDa ectodomain and 40 kDa intracellular domain fragments [8]. Therefore, we next tested whether MMP-7 was responsible for E-cadherin processing in the MDCK and C57MG cells. We observed by immunoprecipitation that the addition of exogenous MMP-7 to confluent MDCK cells resulted in the shedding of the E-cadherin ectodomain into the conditioned media (Figure 4(a)). In confluent MMP-7 expressing C57MG cell lines, we also identified significantly higher levels of the E-cadherin ectodomain in conditioned media compared to the empty vector control cell lines (Figure 4(b)). In agreement with enhanced shedding of the ectodomain, we identified higher levels of the 40 kDa intracellular domain in the MMP-7 expressing cell lines compared to the control (Figure 4(b)). These data demonstrate that MMP-7 mediates E-cadherin processing in nontransformed epithelial cell lines.

### 3.5. Epithelial Cell Proliferation Is Potentiated by MMP-7 and E-Cadherin

**Figure 5:**
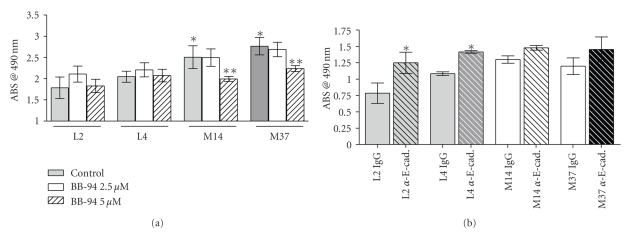
Epithelial proliferation is mediated by MMP-7 processing of E-cadherin. (a) Growth of the C57MG cell lines in the presence or absence of the broad spectrum metalloproteinase inhibitor (BB-94) over a 24-hour period was determined by MTT assay. Asterisk denotes that the proliferation of the MMP-7 expressing cell lines (M14/M37) is significantly higher than the empty vector controls (L2/L4), *P* < .05 while double asterisks denote that proliferation is significantly inhibited in the presence of BB-94 compared to the control conditions, *P* < .05. Significance was determined using ANOVA followed by Tukey's post hoc test. (b) The effect of E-cadherin on cell growth in the absence (IgG) or presence of an E-cadherin blocking antibody (*α*-E-cad) was measured using an MTT assay. Asterisk denotes significantly higher growth in the presence of the E-cadherin blocking antibody in comparison to the IgG treated controls, *P* < .05. Significance was determined using ANOVA followed by Tukey's post hoc test.

Collectively, our data demonstrate that MMP-7 promotes disruption of the adherens junction that in turn results in a loss of epithelial polarization and increased proliferation. Given that MMP-7 can mediate E-cadherin processing, we next examined whether the effects on epithelial cell proliferation were related to the E-cadherin cleavage. We first confirmed that the proliferation phenotype directly resulted from metalloproteinase activity. To this end, the empty vector and MMP-7 expressing C57MG clones were incubated in the presence or absence of the metalloproteinase inhibitor, BB-94. Over 24 hours, we identified that treatment with BB-94 (5 *μ*M) reduced the proliferation of the MMP-7 expressing cell lines to the level of the control empty vector cell lines suggesting that metalloproteinases could promote the proliferation of nontransformed mammary epithelial cells directly as assessed by MTT assay (L2 Control: 1.74 ± 0.25 versus L2 BB-94 5 *μ*M: 1.8 ± 0.15, L4 Control: 2.04 ± 0.13 versus L4 BB-94 5 *μ*M: 2.01 ± 0.15, M14 Control: 2.51 ± 0.27 versus M14 BB-94 5 *μ*M: 1.993 ± 0.06, M37 Control 2.75 ± 0.21 versus M37 BB-94 5 *μ*M: 2.237 ± 0.10 ABS at 490 nm, Figure 5(a)). Of note, BB-94 did not impact the proliferative ability of the empty vector control cell lines (Figure 5(a)), thus suggesting that MMP-7 is the metalloproteinase promoting the proliferative phenotype in these cells. 

To then test whether perturbation of E-cadherin could directly mediate cell proliferation in the C57MG clones, confluent cultures were pretreated with EGTA in order to disrupt the homotypic E-cadherin binding and then returned to normal culture conditions in the presence or absence of an E-cadherin blocking antibody that prevented E-cadherin binding between cells. The results demonstrate that the blocking of E-cadherin significantly promoted the growth of confluent empty vector control C57MG cell lines but had no impact on the growth of the MMP-7 expressing clones compared to respective controls incubated with control antibody (L2 IgG: 0.79 ± 0.06 versus L2 *α*-E-cad: 1.25 ± 0.07, L4 IgG: 1.08 ± 0.03 versus L4 *α*-E-cad: 1.415 ± 0.02, M14 IgG: 1.30 ± 0.03 versus M14 *α*-E-cad: 1.48 ± 0.02, M37 IgG 1.2 ± 0.07 versus M37 *α*-E-cad: 1.45 ± 0.10 ABS at 490 nm, Figure 5(b)). Thus, MMP-7 expression rendered the E-cadherin blocking antibody impotent, indicating that E-cadherin is downstream of MMP-7.

Overall, these data demonstrate that MMP-7 can promote cell proliferation and that the molecular mechanism underlying this observation is due, in part, to the perturbation of E-cadherin mediated cell-cell contact.

### 3.6. E-Cadherin Mediates Epithelial Cell Proliferation in a RhoA Dependent Manner

**Figure 6:**
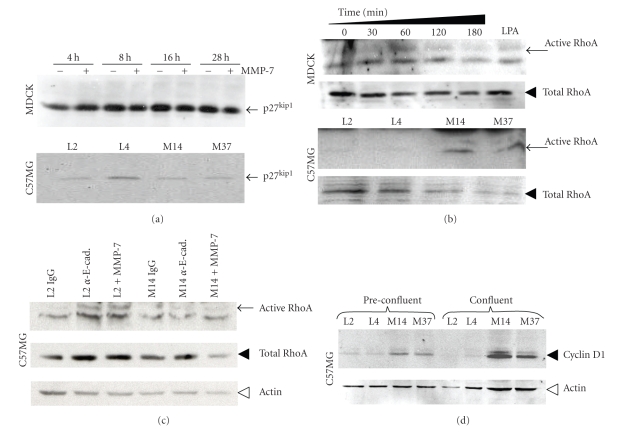
MMP-7 cleavage of E-cadherin does not affect p27^kip-1^ levels but enhances RhoA activity. (a) Change in p27^kip-1^ levels in polarized MDCK cells over time (hours, h) in the absence (−) or presence (+) of exogenous MMP-7 (100 ng/mL) was assessed by immunoblot analysis of the cell lysates (upper panel). p27^kip-1^ levels (arrow) in cell extracts of confluent vector control and MMP-7 expressing cell lines were also examined (lower panel). (b) Change in confluent-polarized MDCK RhoA activity in response to the addition of exogenous MMP-7 (100 ng/mL) over time (minutes, min) was examined using a Rhotekin pull down assay as described in the materials and methods followed by immunoblot analysis for active RhoA. Direct immunoblot analysis for total RhoA served as a control for loading. Arrow and arrowhead indicate active and total RhoA, respectively. LPA was used as a positive control for RhoA activity. The level of active RhoA in confluent vector control and MMP-7 expressing cell lines was also determined. (c) RhoA activity (arrow) in lysates obtained from confluent empty vector control and MMP-7 expressing cell lines treated either with IgG control or with E-cadherin blocking (*α*-E-cad) antibodies in addition to exogenous MMP-7 (100 ng/mL) for 1 hour was examined using the rhotekin pull down assay followed by immunoblot analysis of RhoA (upper panel). Total RhoA (arrowhead) and actin (open arrow head) levels were examined by direct immunoblot analysis of the cell lysates and served as a loading control. (d) Analysis of cyclin D1 (arrowhead) levels in whole cell lysates derived from preconfluent and confluent empty vector control and MMP-7 expressing cell lines. Actin was used as a loading control (open arrowhead).

Our data suggest that MMP-7 processing of E-cadherin mediated the proliferation of nontransformed epithelial cells. Next, we examined the precise molecular mechanism through which E-cadherin mediated this effect. Previous reports have identified that E-cadherin can regulate cell cycle progression via negative regulation of the cell cycle inhibitor, p27^kip1^ [28]. However, analysis of lysates derived from control and MMP-7-treated MDCK cells and from the empty vector control and MMP-7 expressing mammary epithelial cells revealed no major differences in the levels of p27^kip1^ (Figure 6(a)). In separate experiments, no changes in cytosolic versus nuclear p27^kip-1^ were observed (data not shown). Several studies have tied RhoA activity status to E-cadherin function [29–35], which prompted us to analyze the activity of RhoA in both the MDCK cells after treatment with exogenous MMP-7, and in the C57MG control and MMP-7-expressing clones. We detected heightened RhoA activity (normalized to total RhoA) compared to controls in both cases (Figure 6(b)). We then used the C57MG clones to test whether the enhanced RhoA activity was downstream of E-cadherin. In the C57MG empty vector control cell line, blocking E-cadherin homotypic binding via the addition of blocking E-cadherin antibodies significantly enhanced RhoA activation in comparison to IgG-treated controls (Figure 6(c)). An identical result was achieved when control cells were treated with recombinant active MMP-7. As expected, in cells already expressing MMP-7 in which E-cadherin is already perturbed, neither blocking antibody nor addition of exogenous MMP-7 had any effect on RhoA activity (Figure 6(c)). Thus, perturbation of E-cadherin by either antibody blockade or MMP-7-mediated processing results in the activation of RhoA. 

RhoA activity has long been implicated in regulation of cell cycle progression by multiple pathways including increased levels of cyclin D1 [36]. Since p27^kip1^ levels were unchanged, we therefore examined the levels of cyclin D1 in the C57MG control and MMP-7-expressing clones. Our data demonstrate that in both preconfluent and confluent C57MG cell lines, MMP-7 expression is associated with enhanced levels of cyclin D1 (Figure 6(d)). Collectively, these data suggest that MMP-7 processing of E-cadherin in nontransformed cells leads to the perturbation of the adherens junction, increased RhoA activity, and higher levels of cyclin D1 expression that in turn result in enhanced proliferation. 

## 4. Discussion

Unchecked cell proliferation is a hallmark of epithelial tumorigenicity and tumor progression. Identification of the mechanisms that regulate how epithelial cells proliferate and grow is key to understanding the epigenetic changes that drive cancer. The formation of tight adherens junctions between epithelial cells is essential for proper function of the epithelium. Studies have identified that disruption of the adherens junction by proteinases in epithelial cells can lead to a more migratory function but it is unclear if the perturbation of the cell-cell contact machinery can also lead to proliferation in nontransformed epithelial cells [8, 16, 17]. In the current study we have shown that inhibition of the function of a critical component of the adherens junction, E-cadherin, via proteolytic processing by MMP-7 promotes epithelial cell proliferation via enhanced RhoA activity and cyclinD1 expression. 

In addition to its well-understood adhesive function, E-cadherin is an important regulator of cell signaling [37, 38]. Catenins, the other main components of adherens junctions, are important signaling molecules whose activity is modulated by their interaction with E-cadherin. For example, *β*-catenin when not bound by E-cadherin can participate in Wnt signaling [38], and p120-catenin is a critical regulator of Rho and Rac-GTPases [39]. Notably, E-cadherin blockade by neutralizing antibody does not lead to dissociation of *β*-catenin from the adherens junctions [23]. We also saw no significant changes in *β*-catenin by western blot analysis after exposing our cells to MMP-7 (data not shown). A likely explanation is that in these nontransformed cells, without a Wnt signal, any *β*-catenin released from the junctions will be ubiquitinated and degraded. Recent evidence suggests that both intra- and extracellular fragments of E-cadherin itself participate in signaling directly through activation of ErbB receptors [18] and nuclear activity of the p120/kaiso complex [40], respectively. A well-studied consequence of E-cadherin-regulated signaling is proliferation. One method by which this occurs is via upregulation of the cell cycle inhibitor p27^kip1^, which has been described as a major mechanism of contact inhibition [23]. However, it is clear that the same pathways are not operational in all cells. Indeed, a recent report suggests that E-cadherin and p27^kip1^ actually have an inverse relationship in renal and other cancer cells, so that increased E-cadherin is associated with reduced p27^kip1^ [41]. In the two cell lines used in this report, renal MDCK cells and mammary C57MG cells, there was no apparent change in p27^kip1^ levels following E-cadherin cleavage. Instead, RhoA activation was seen as a direct result of E-cadherin perturbation either by blocking antibodies or MMP-7 cleavage. Changes in Rho-GTPase activity have previously been associated with loss of E-cadherin adhesive function [29–31, 33]. Unsurprisingly, the activation of RhoA can contribute to the migratory phenotype via control of the actin cytoskeleton [9, 31]. However, RhoA activation has also long been known to influence cell cycle progression [36]. This can occur via promotion of the degradation of cell cycle inhibitors such as p21 and p27^kip1^ or via increased gene expression of cell cycle promoters such as cyclin D1 [36, 42–44]. In our cell lines, increased cyclinD1 seems to be the major result of the MMP-7-initiated E-cadherin/Rho A activation pathway.

Our data identify that MMP-7 mediates cell proliferation via the processing of E-cadherin in nontransformed epithelial cells, a conclusion that is supported by our data using E-cadherin blocking antibodies (Figure 5). However, the possibility that MMP-7 can promote proliferation via other mechanisms is also likely given that the proteinase has a wide variety of substrates [2]. In fact we have recently shown that under three-dimensional (3D) culture conditions, MMP-7 processing of heparin bound epidermal growth factor in the C57MG cells is responsible for promoting cell proliferation via the ErbB4 receptor [4]. Intriguingly, this effect is not apparent under 2D conditions. Therefore, *in vivo* it is plausible that MMP-7 expression results in the initiation of a cellular program that cumulatively is responsible for the initiation of proliferation in normal epithelial cells. This supposition of MMP-7 as an effector of this program is further supported by studies demonstrating how E-cadherin and ErbB receptors work in concert to orchestrate proliferation [45, 46]. 

In previous studies, we identified that MMP-7 processing of E-cadherin leads to enhanced cell migration [8]. Furthermore, we observed that the ectodomain of E-cadherin was responsible for promoting the migratory phenotype since the immunodepletion of the soluble ectodomain of E-cadherin reestablished E-cadherin function. The results of the current study using a nontransformed mammary gland epithelial cell line are in agreement with the concept that MMP processing of E-cadherin leads to the disruption of the adherens junction and the induction of cell migration. However, it is possible that the soluble ectodomain of E-cadherin may mediate other cellular effects. For example, the ectomain fragment released by a disintegrin and metalloproteinase-15 (ADAM-15) processing of E-cadherin can bind to the ErbB receptors, ErbB2 and ErbB3, and stimulate proliferation. The approximate molecular weights of the E-cadherin ectodomains generated by MMP-7 and ADAM-15 are similar in size; however it is possible that variations in the precise amino acid sequence at the cleavage site may dictate the biological effects of the generated E-cadherin ectodomain [18]. Furthermore, we and others have also shown that E-cadherin is susceptible to processing by MMP-3 while others have shown that ADAM-10, cathepsins, and serine proteases can also promote E-cadherin processing [8, 16, 17, 19–22]. Therefore, whether the processing of E-cadherin by proteinases is a means of regulating specific E-cadherin functions or represents a substrate overlap between proteinases requires further investigation. Nevertheless, our studies and others linking E-cadherin processing to migration and proliferation support the role of MMP-7 in initiating a cell proliferation program in nontransformed epithelial cell lines. 

Interestingly, our data identify that the addition of exogenous MMP-7 to the basolateral rather than the apical surface of the MDCK cells results in the processing of E-cadherin and subsequent increased proliferation. This observation appears contrary to our earlier studies in the MDCK cell line that indicated overexpression of MMP-7 promoted proliferation via an apical but not basolateral pathway [7]. In that study however, the overexpressed MMP-7 was in the pro- or latent form. Conversion to the active enzyme was only evident at the apical surface which would explain why no effects were seen at the basal surface. However, our observation here that when active MMP-7 interacts with proteins on the basolateral surfaces, a proliferative effect can be seen suggests that the expression of MMP-7 by stromal cells may impact the behavior of the normal epithelium. While MMP-7 is often expressed by glandular epithelium, it has also been shown to be highly expressed by macrophages and osteoclasts [47–49]. In addition, we have previously reported that basement membrane integrity is disrupted in transgenic mice overexpressing MMP-7 in the mammary epithelium suggesting that this MMP can also impact the integrity of the basolateral compartment [6]. Therefore, it is possible that in the context of inflammation or infection, MMP-7 secreted from other cellular sources may reach the basolateral compartment of the epithelium and promote epithelial cell migration and proliferation. 

In addition to identifying roles for E-cadherin in the proliferation of nontransformed epithelial cells, the findings of the current study also have implications for pathological disease. For example, MMP-7 is often expressed by glandular epithelium including those of the breast and colon [49], and we have previously identified that MMP-7 can promote mammary gland and colon tumorigenesis [50, 51]. Therefore, given the findings of the current study, it is plausible that the aberrant expression of MMP-7 could result in the initiation of a cellular program that promotes epithelial proliferation and migration, and that MMP-7 processing of E-cadherin is a molecular effector of this program.
